# Neupogen and mesenchymal stem cells are the novel therapeutic agents in regeneration of induced endometrial fibrosis in experimental rats

**DOI:** 10.1042/BSR20170794

**Published:** 2017-10-11

**Authors:** Dina Sabry, Abeer Mostafa, Samar Marzouk, Walaa Ibrahim, Hanan H.M. Ali, Aymen Hassan, Ashraf Shamaa

**Affiliations:** 1 Medical Biochemistry and Molecular Biology department, Cairo University,Cairo, Egypt; 2 Pathology Department, Cairo University, Cairo, Egypt; 3 Gynecology and Obstetric Department, Faculty of Medicine, Cairo University, Cairo, Egypt; 4 Surgery, Anaesthesiology and Radiology Department, Faculty of Veterinary Medicine, Cairo University, Cairo, Egypt

**Keywords:** endometrial fibrosis, mesenchymal stem cell, Neuopogen

## Abstract

Endometrial fibrosis is the presence of intrauterine adhesions (IUAs) after any uterine surgery or curettage and it results in infertility and recurrent pregnancy loss. We evaluated the role of human mesenchymal stem cells (hMSCs) as a therapeutic agent of endometrial fibrosis. We also compared the effect of MSCs with the effect of estrogen and neupogen either each alone or as a combined therapy with MSCs. This experimental study was performed on 84 albino rats which were divided into seven groups (*n*=12 rats/group) as follows, *group*1: normal control rats, *group* 2: induced fibrosis, *group* 3: induced fibrosis that received oral estrogen, *group* 4: induced fibrosis that received hMSCs, *group* 5: induced fibrosis that received hMSCs and estrogen, *group* 6: induced fibrosis that received neupogen, and *group* 7: induced fibrosis that received hMSCs and neupogen. The extent of fibrosis, vascularization, and inflammation were evaluated by; qRT-PCR for interleukin 1 (IL-1), interleukin 6 (IL-6), TNF, vascular endothelial growth factor (VEGF), transforming growth factor-β (TGF-β), and RUNX; ELISA for connective tissue growth factor (CTGF); Western blotting for collagen-I; immunohistochemistry examination for VEGF and RUNX-2; and histopathological assessment. In therapeutic groups either by hMSCs alone or combined with estrogen or neupogen; fibrosis and inflammation (IL-1, IL-6, TNF, TGF-β, RUNX, CTGF, and collagen-I) were significantly decreased but vascularization (VEGF) was significantly increased (*P*<0.05) compared with induced fibrosis group. The most significant result was obtained in fibrosis that received combined therapy of hMSCs and neupogen (*P*=0.000). Stem cells and neupogen are a highly effective alternative regenerative agents in endometrial fibrosis.

## Introduction

Endometrial fibrosis is the presence of intrauterine adhesion (IUA), which may follow uterine surgery, e.g. curettage done after miscarriage or due to infections such as TB [1]. In 2013, it was estimated that 171 million women were affected [2]. The fibrosis is progressive and a chronic process. It results from interaction of inflammatory cytokines such as interleukin-1 (IL-1), interleukin 6 (IL-6), TNF, and fibrotic factors as TGF, connective tissue growth factor (CTGF), and collagen 1. This interaction allows excessive accumulation of ECM, which is a key step in fibrosis [3]. Traditional treatment of IUA is surgical removal of the adhesion followed by hormonal therapy. Estrogen is the most commonly used hormone in supraphysiological dose to allow the regeneration of the endometrium. However, this modality of treatment is characterized by high failure rate due to reformation of the adhesion [4]. Mesenchymal stem cells (MSCs) provide a novel method in the treatment of fibrotic disease due to their ability to evade the immune detection, secrete anti-inflammatory and antifibrotic mediators [5]. Neupogen is a granulocyte colony-stimulating factor (G-CSF) analog used to stimulate the proliferation and differentiation of granulocytes and release stem cells into the bloodstream [6]. The aim of this work is to investigate the effect of estrogen, neupogen, and MSCs separately or as a combined therapy in regeneration of induced endometrial fibrosis in rats.

## Material and methods

### Isolation of human umbilical cord MSCs

Human umbilical cord specimens were obtained using protocols approved by the Ethical Committee of Faculty of Medicine, Cairo University, in collaboration with the Labor and Delivery nursing staff. After obtaining patients’ informed consents, four fresh cord samples of women with healthy pregnancies were retrieved during casarean deliveries. Wharton jelly was harvested from term deliveries at the time of birth. Wharton jelly was minced and incubated with collagenase II enzyme (IgG, *C. histoliticum*, Biological life science, U.S.A.) at 37°C for 2 h. Strainer (Invitrogen, CA, U.S.A.) was applied to remove tissue debris. Isolated cells were cultured and propagated in 10% and 1× pen/strep (Invitrogen, CA, U.S.A.) at 5% CO_2_, 37°C until reaching 70–80% confluence. After 1 week of culture; cells were washed with PBS and trypsinized with 0.25% trypsin for 5 min at 37°C. After centrifugation, cell pellets were resuspended and propagated in RPMI-160 medium as first-passage cultures [7]. For MSCs characterization; 1 × 10^5^ cells were incubated with 10 μl of monoclonal antibodies: CD105 PE, CD29 PE, and CD34 PE (Beckman Coulter, U.S.A.) at 4°C in the dark, same species isotypes served as a negative control. After incubation, 2 ml of PBS containing 2% FBS solution were added, centrifuged, and cells were resuspended with PBS. FACS analysis was performed using CYTOMICS FC 500 (Beckman Coulter, FL, U.S.A.) and CXP Software version 2.2 for interpretation. MSCs were further characterized by their differentiation into adipocytes. The differentiation was achieved by adipocytes StemPro® adipogenesis differentiation kit (Gibco, Life Technology) and they were stained by Oil Red O stain (Sigma, catalog# 0-0625). MSCs were labeled with GFP (pAcGFP1-N1 vector, Clontech Laboratories, Inc. (U.S.A.), catalog# 632469) for *in vivo* tracing and observed in unstained uterine tissues cryosections using Fluorescence Microscope (Leica Microsystems CMS GmbH, Ernst-Leitz-Straße, Wetzlar D-35578, Germany).

### Experimental animals

An approval from Institutional Animal Care was taken prior to study. The Animal House Unit of Cairo University provided the veterinary care. Preparation of the experimental animal model of induced endometrial fibrosis was done. Under flutothane inhalational anesthesia, laparotomy was done and the pelvic region was exposed. Then, 0.1 ml of 10% trichloroacetic acid was injected into right uterine horn. One month after surgery, two rats were killed to confirm the induction of endometrial fibrosis [8]. Then, after establishing the model, the study started, with 84 female rats, recruited from the animal house at Faculty of Medicine, Cairo University. The average weight of the animals was 170–230 g. The animals were housed in wire mesh cages at room temperature with 12:12-h light-dark cycles and were maintained on standard rat chow and tap water. Animals were randomly divided into seven groups (12 animals each): *group* 1: a negative control; *group* 2: induced endometrial fibrosis (pathological control); *group* 3: induced endometrial fibrosis that received human mesenchymal stem cells (hMSCs) (2 × 10^6^ cells/ml intraperitoneally/week) [8]; *group* 4: induced endometrial fibrosis that received 0.1 mg/kg daily oral estrogen [8], *group* 5: induced endometrial fibrosis that received 2 × 10^6^ hMSCs + 0.1 mg/kg and daily oral estrogen; *group* 6: induced endometrial fibrosis that received neupogen (300 µg/ml IV injection in tail vein, three times/week) [9], and *group* 7: induced endometrial fibrosis that received 2 × 10^6^ hMSCs + 300 µg/ml neupogen.

After 1 month of stem cell and drugs administration, the rats were killed and uterine tissues were harvested and subjected to histopathological and immunhistochemical evaluations and molecular study.

### Histopathological evaluation

Uterine tissues of all the studied groups were separately collected and fixed overnight in 40 g/l paraformaldehyde at 4°C. The uterine horns were cut by serial transverse sections and put into processing cassette. Then, the paraffin blocks were prepared from each cassette separately. In brief, tissue dehydration carried out in ascending concentrations of ethanol (alcohol) with 70, 90, and 100% (three changes). Then, specimens were cleared by three changes of xylene. Thereafter, the specimens are ready to be infiltrated by wax and formation of paraffin blocks. Two slides of 4-μm thick sections were prepared: one for routine Hematoxylin–Eosin (H&E) staining and the other for Masson’s trichrome stain (for highlighting fibrosis). Then, these slides were examined for fibrosis, inflammation, and vascular proliferation graded on a semiquantitative, Table 1 [8].

**Table 1:** A modified semiquantitative histopathological scale for uterine tissue grades

Grades	Fibrosis	Inflammation	Vasculature	Uterine gland
Grade 0	No fibrosis	No inflammation	No vascular proliferation	No glandular proliferation
Grade 1	Minimal loose fibrosis	Presence of occasional lymphocytes and plasma cells	Mild vascular proliferation	Mild glandular proliferation
Grade 2	Moderate fibrosis	Presence of plasma cells, eosinophils, and neutrophils	Moderate vascular proliferation	Moderate glandular proliferation
Grade 3	Dense fibrosis	Presence of many inflammatory cells and microabscesses	Intense vascular proliferation	Intense glandular proliferation

### Immunhistochemical evaluation

After slide preparation as discussed above, antigen retrieval was performed using microwave heating (three times for 10 min in 10 mM citrate buffer, pH: 6.0) after inhibition of endogenous peroxidase for 15 min. The slides were incubated for 1 h with rabbit polyclonal antibodies to vascular endothelial growth factor (VEGF) (VEGF antibody (VG1) Novus Biologicals (NB100-664)), RUNX (RUNX2/CBFA1 antibody) Novus Biologicals (cat# L012V1) at room temperature, then washed using PBS and incubated with secondary antibody (Invitrogen, U.K.) for 15 min followed by PBS wash. Finally, the detection of bound antibody was accomplished using the avidin–biotin complex (ABC) reagent for 20 min, then PBS wash. A 0.1%-solution of diaminobenzidine (DAB) (Thermo Scientific, U.S.A.) was used for 5 min as a chromogen. Slides were counterstained with Mayer’s Hematoxylin for 5–10 min. All histopathological examinations were performed by a designated pathologist experienced in rat histology. To evaluate VEGF expression, the number of capillary vessels and proliferation cells were counted and averaged from at least four randomly selected fields under a magnification of 200×. For RUNX, the average optical density (mean density) represented the intensity of protein expression and was counted in four random fields under a magnification of 200×.

### Quantitative RT-PCR

Real-time PCR was performed for quantitative genes expression of IL-1, interleukin 6 (IL-6), tumor necrosis factor-α (TNF-α), VEGF, transforming growth factor-β (TGF-β), and Runt-related genes (RUNIX). Uterine samples of all the studied groups were lysed and total RNA was isolated with GeneJET Kit (Thermo Fisher Scientific Inc., Germany, #K0732). Ten nanograms of the total RNA from each sample were used for reverse transcription with subsequent amplification with Bioline, a median life science company, U.K. (SensiFAST™ SYBR® Hi-ROX) One-step Kit (catalog number PI-50217 V) in a 48-well plate using the Step-one instrument (Applied Biosystems, U.S.A.). Thermal profile was as follows: 45°C for 15 min in one cycle (for cDNA synthesis), 10 min at 95°C for reverse transcriptase enzyme inactivation, followed by 40 cycles of PCR amplification. Each cycle was carried out for : 10 s at 95°C, 30 s at 60°C, and 30 s at 72°C. Changes in the expression of each target gene were normalized relative to the mean critical threshold (*C*_T_) values of GAPDH as the housekeeping gene by the ΔΔ*C*_t_ method. Primers’ sequences for all the studied genes were demonstrated in Table 2.

**Table 2:** Primers sequences of all the studied genes

Genes	Primer sequence from 5′ to 3′	GenBank accession number
*TGF-β*	Forward: CTACTGCTTCAGCTCCACAG	XM016135677.1
	Reverse: GCACTTGCAGGAGCGCAC	
*TNF-α*	Forward: GCCTCTTCTCATTCCTGCTT	AF269160.1
	Reverse: CACTTGGTGGTTTGCTACGA	
*VEGF*	Forward: CTCCGAAACCATGAACTTTCTGCTC	NM031836.2
	Reverse: CAGCCTGGCTCACCGCCTTGGCTT	
*IL-1*	Forward: CTGTGGCAGCTACCTATGTCTTG	NM031512.2
	Reverse: AGGTCGTCATCATCCCACGAG	
*IL-6*	Forward: TTCCATCCAGTTGCCTTCTT	NM001314054.1
	Reverse: ATTTCCACGATTTCCCAGAG	
*RUNX*	Forward: CCAGATGGGACTGTGGTTACC	XR385272.3
	Reverse: ACTTGGTGCAGAGTTCAGGG	
*GAPDH*	Forward: ACAGTCCATGCCATCACTGCC	NG009348.3
	Reverse: GCCTGCTTCACCACCTTCTTG	

### ELISA

Tissue CTGF was assessed to evaluate the fibrosis extent in uterine tissue of all the studied groups (pg/ml). Rat CTGF ELISA Kit (Elabscience Biotechnology Co., Ltd, Wuhan, Hubei Province, catalog number E-EL-R0259) was used according to manual’s instructions.

### Western blot

The collagen type І antibody used was purchased from Thermo Scientific (MA1-26771)**.**

Protein from uterine tissue was extracted by RIPA lysis buffer which was provided by Bio Basic Inc. (Markham, Ontario L3R 8T4, Canada). Extracted protein was separated by SDS/PAGE on 4–20% polyacrylamide gradient gels. After incubation in 5% non-fat dry milk, Tris/HCl, 0.1% Tween 20 for 1 h; collagen-I monoclonal antibody was added to one of the membranes containing specimen samples and incubated at 4°C overnight. Appropriate secondary antibodies were incubated for 2 h at room temperature. After being washed twice with 1× TBST, densitometric analyses of the immunoblots were performed to quantitate the amount of collagen-I against control sample by total protein normalization using image analysis software on the ChemiDoc MP imaging system (version 3) produced by Bio–Rad (Hercules, CA).

### Statistical analysis

Data were coded and entered using the statistical package SPSS version 22. Data were summarized using mean and S.D. Comparisons between groups were done using ANOVA with multiple comparisons post-hoc test [10]. *P*-values less than 0.05 were considered as statistically significant**.** Data were expressed as mean ± S.D., *P*-value <0.05 was significant. *: statistically significant compared with corresponding value in normal control group. ^#^: statistically significant compared with corresponding value in fibrosis group °: statistically significant compared with corresponding value in fibrosis and estrogen group ^@^: statistically significant compared with corresponding value in fibrosis and MSCs group. ^$^: Statistically significant compared with corresponding value in fibrosis and neupogen group. ▪: statistically significant compared with corresponding value in fibrosis + MSCs + estrogen group.

## Results

**Figure 1:**
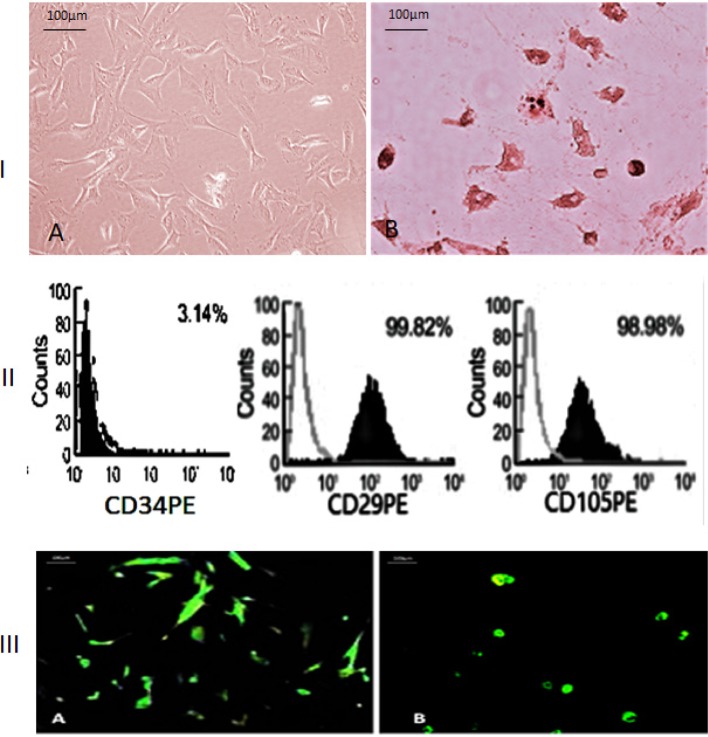
MSCs were assessed for propagation, differentiation, characterization, and labeling Panel **I**(A): MSCs were isolated as fibroblast-like cells. (B) MSCs were differentiated into adipocytes stained with Oil Red O stain. Panel **II**: FACS analysis characterized MSCs; they were positive for CD29 and CD105 surface markers and negative for CD34. Panel **III**(A) MSCs were labeled with GFP *in vitro*. (B) Uterine tissue showed *in vivo* GFP-labeled cells for tracing of MSCs in uterine tissue.

The MSCs in culture were identified morphologically by assessing fusiform fibroblast like cells (Figure 1, panel I(A)), and then differentiated into adipocytes (Figure 1, panel I(B)). The differentiated adipocytes were stained with Oil Red O stain. MSCs were further characterized by cell surface phenotyping assessment. MSCs showed 99.82% positive expression for the β1-integrin CD29, 98.98% positive expression for the endoglin receptor CD105, and negative for CD 34 (Figure 1, panel II). MSCs were labeled in culture with GFP (Figure 1, panel III(A)). GFP-labeled MSCs were traced in uterine tissue (Figure 1, panel III(B)).

**Figure 2.1:**
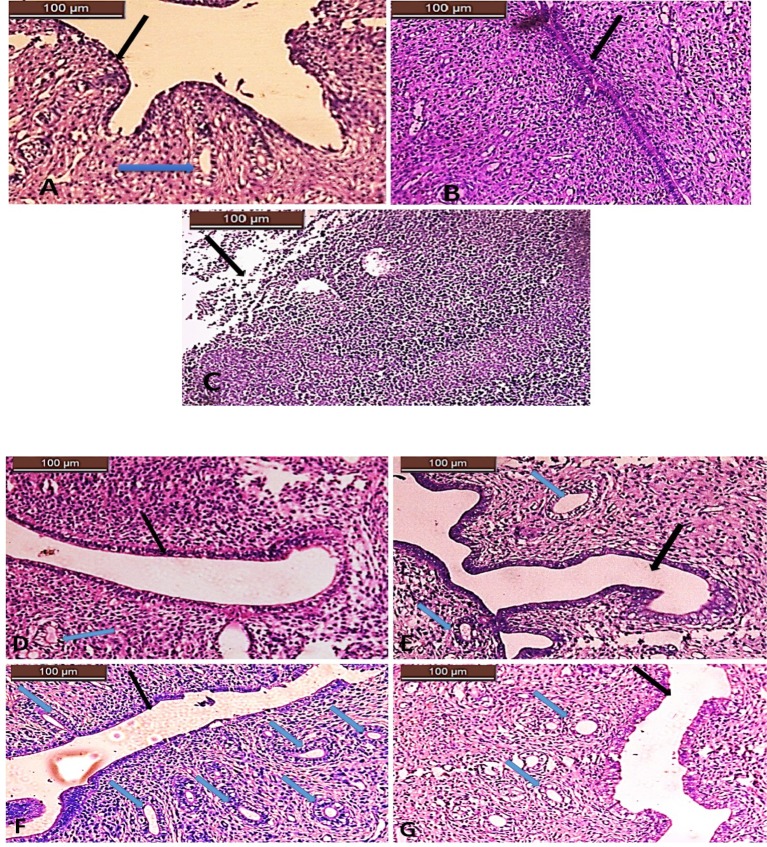
Histopathological assessment of uterine tissues in all the studied groups (**A**) Normal uterus, (**B**) induced endomertial fibrosis, (**C**) fibrosis treated with estrogen only, (**D**) fibrosis treated with MSCs only, (**E**) fibrosis treated with estrogen and MSCs, (**F**) fibrosis treated with neupogen and MSCs, and (**G**) fibrosis treated with neupogen only. *Black arrow* represents the uterine cavity and *blue arrow* the uterine gland.

The uterine specimens were evaluated histopathologically for fibrosis, inflammation, vascular, and uterine glands proliferation according to grades in Table 1 The normal uterine tissue presented with intact endothelium and patent uterine cavity by the *black arrow*, no inflammatory cells (grade 0), normal vasculature, and normal uterine gland by the *blue arrow* (Figure 2.1A). The induced endometrial fibrosis presented with obliterated uterine cavity, grade 3 dense fibrosis, grade 2 inflammation, grade 1 mild vascular proliferation, and grade 0 no glandular proliferation (Figure 2.1B). The induced endometrial fibrosis treated with estrogen only showed necrotic endothelium, grade 3 dense fibrosis, grade 3 inflammation, grade 1 mild vascular proliferation, and grade 0 no glandular proliferation (Figure 2.1C). The induced endometrial fibrosis treated with stem cells only showed patent uterine cavity, grade 2 moderate fibrosis, grade 2 inflammation, grade 2 moderate vascular proliferation, and grade 2 moderate glandular proliferation (Figure 2.1D). The induced endometrial fibrosis treated with stem cells and estrogen with patent uterine cavity, grade 1 mild fibrosis, grade 2 inflammation, grade 2 moderate vascular proliferation, and grade 2 moderate glandular proliferation (Figure 2.1E). The induced endometrial fibrosis treated with stem cells and neupogen presented with patent uterine cavity, grade 0 no fibrosis, grade 0 no inflammation, grade 3 intense vascular proliferation, and grade 3 intense glandular proliferation(Figure 2.1F). The induced endometrial fibrosis treated with neupogen only were having patent uterine cavity, grade 1 mild fibrosis, grade 2 inflammation, grade 2 moderate vascular proliferation, and grade 2 moderate glandular proliferation (Figure 2.1G).

**Figure 2.2:**
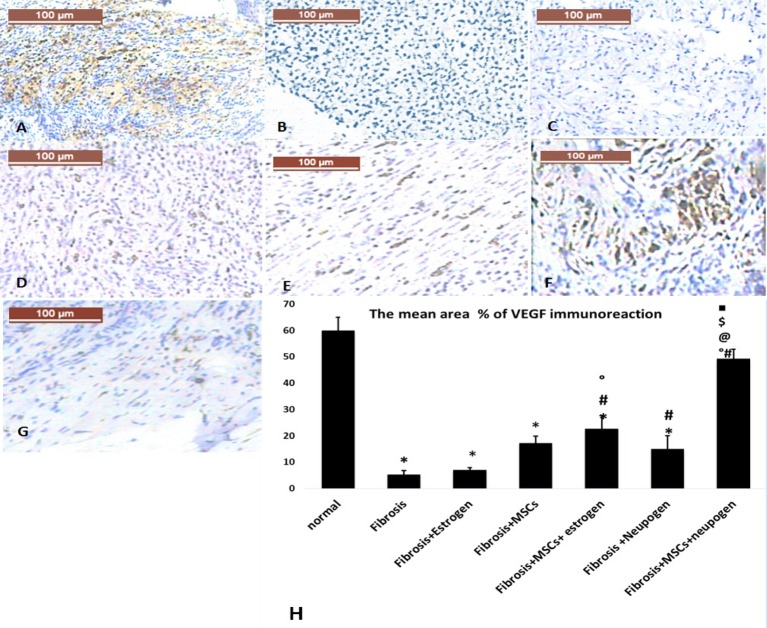
The immunohistochemical assessment for VEGF of uterine tissues in the all studied groups (**A**) Normal uterus, (**B**) induced endomertial fibrosis, (**C**) fibrosis treated with estrogen only, (**D**) fibrosis treated with MSCs only, (**E**) fibrosis treated with estrogen and MSCs, (**F**) fibrosis treated with neupogen and MSCs, and (**G**) fibrosis treated with neupogen only. (**H**) Mean percent area for VEGF positive immune reaction.

**Figure 2.3:**
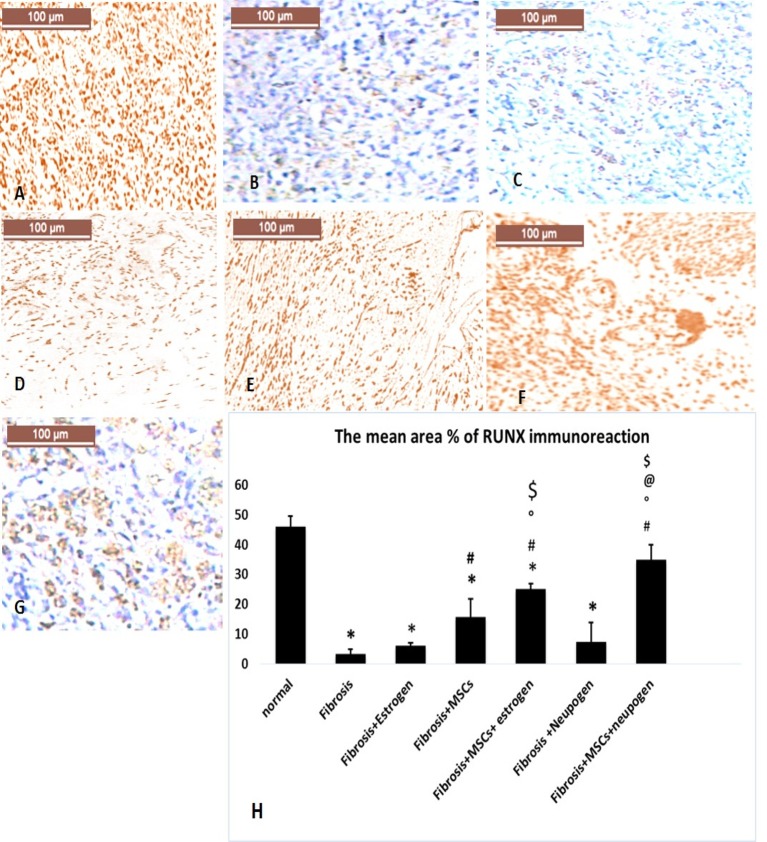
The immunohistochemical assessment for RUNX of uterine tissues in all the studied groups (**A**) Normal uterus, (**B**) induced endomertial fibrosis, (**C**) fibrosis treated with estrogen only, (**D**) fibrosis treated with MSCs only, (**E**) fibrosis treated with estrogen and MSCs, (**F**) fibrosis treated with neupogen and MSCs, and (**G**) fibrosis treated with neupogen only. (**H**) Mean percent area for RUNX positive immune reaction.

Immunhistochemical staining of VEGF and RUNX were assessed by the presence of brown nuclear positive reactivity (Figure 2.2,2.3), respectively. Staining index was increased in uterine tissue of combined MSCs-treated groups with the highest significant index increase in MSCs + neupogen-treated group when compared with other groups.

**Figure 3:**
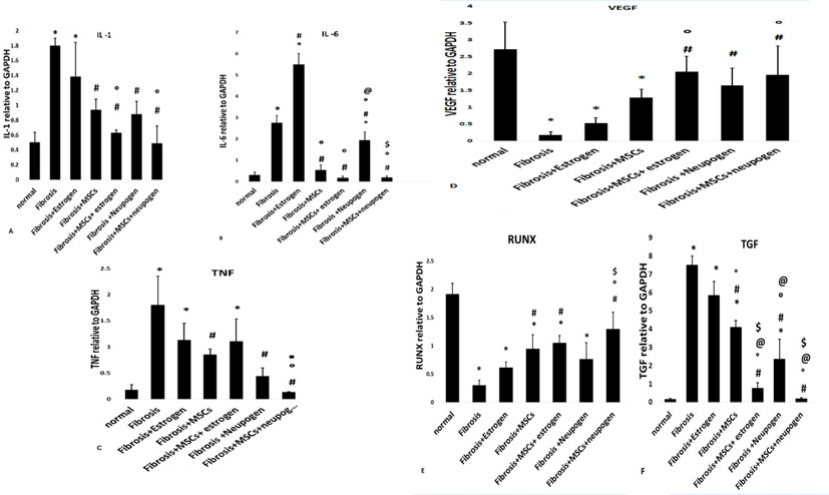
Quantitative genes’ expression of the target genes in all the studied groups (**A**) IL-1, (**B**) IL-6, (**C**) TNF-α, (**D**) VEGF, (**E**) RUNX, and (**F**) TGF-β.

Genes’ expression of inflammatory cytokines (IL-1, IL-6, and TNF-α) showed statistical significant decrease in groups treated either with neupogen only or MSCs only (*P*<0.05), with highly significant decrease observed in MSCs and neupogen treated group compared with fibrosis group (*P*=0.000) (Figure 3A–C, respectively). VEGF expression level has a statistical significant increase in either MSCs treated groups or neupogen-treated group (*P*-value <0.05), with highest significant increase in MSCs and neupogen treated group compared with fibrosis group (*P*=0.015) (Figure 3D).

RUNX as an antifibrotic factor showed a statistical significant increase in MSCs treated group (*P*-value <0.05), with highly significant increase observed in MSCs and neupogen treated group compared with fibrosis group (*P*=0.001) (Figure 3E).

**Figure 4:**
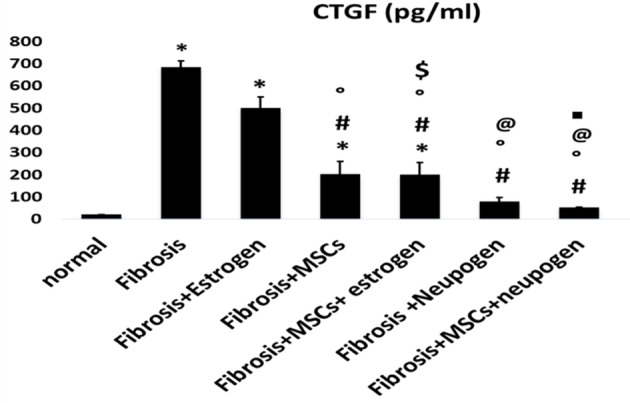
CTGF expression levels in all the studied groups

**Figure 5:**
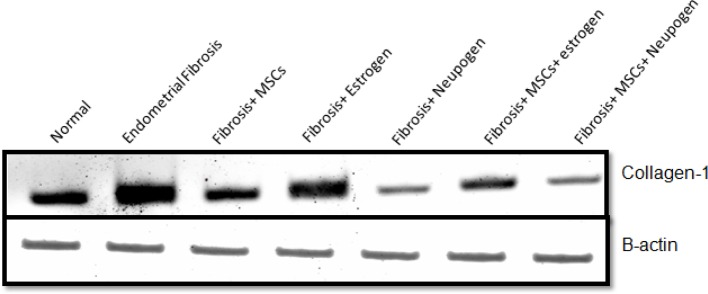
The quantitative scanning densitometry results of collagen-1 normalized compared with β-actin protein levels in different groups

As regarding markers of fibrosis (TGF-β, CTGF, and collagen-I), there was a statistical significant decrease in neupogen treated group and MSCs treated groups (*P*-value <0.05) with greater significant decrease observed in MSCs and neupogen treated group compared with fibrosis group (*P*=0.000) (Figures 3F,4,5 matched with Table 3, respectively).

**Table 3:** Quantitative Western blot protein expression level of collagen-1 in all the studied groups

Groups	Normal	Fibrosis	Fibrosis + MSCs	Fibrosis + estrogen	Fibrosis + neupogen	Fibrosis + MSCs + estrogen	Fibrosis + MSCs + neupogen
**Collagen-1**	0.29 ± 0.05	1.60 ± 0.45*	1.21 ± 0.05*	1.59 ± 0.09*	0.34 ± 0.08^#^°^@^	0.81 ± 0.09**^#^**°	0.20 ± 0.05**^#^**°**^@▪^**

*: statistically significant compared with corresponding value in normal control group. #: statistically significant compared with corresponding value in fibrosis group °: statistically significant compared with corresponding value in fibrosis and estrogen group @: statistically significant compared with corresponding value in fibrosis and MSCs group. $: Statistically significant compared with corresponding value in fibrosis and neupogen group. ▪: statistically significant compared with corresponding value in fibrosis + MSCs + estrogen group.

No statistical significant effect of estrogen treatment compared with fibrosis group regarding either inflammatory cytokines or fibrotic factor. Thus, treatment with estrogen alone is not sufficiently an effective therapy.

## Discussion

IUAs prevent the endometrium from growing, resulting in infertility. Many therapies have been attempted for these conditions, but none have proved to be effective [11]. The general idea is to encourage fast growth of any residual endometrium immediately after surgical removal of the adhesion with the dual purpose of preventing new scar formation and restoring a normal uterine environment. It is supposed that this goal can only be achieved with supraphysiological hormonal levels [12]. However, the efficacy is not same in every patient. Regenerative medicine offers the potential for replacement or repair of different types of cells within damaged tissues. In this work, estrogen therapy, stem cell releasing factor (neupogen) as well as MSCs were evaluated and compared regarding their efficacy in treatment of endometrial fibrosis. The current results revealed that there was a significant decrease in *IL-1* gene expression level in all MSCs treated groups and the neupogen treated group compared with the fibrosis group. *IL-1* gene expression and its relation with development of fibrosis have been stated in previous studies as it promotes hepatic stellate cell (HSC) proliferation, which plays a critical role in development of liver fibrosis and cirrhosis [13]. Similarly, systemic infusion of BMSCs reduced skin contracture, thickening, collagen deposition, and decreased expression of IL-1 in the irradiated skin [14]. In another study where, a model of endometriosis was established and up-regulation of fibrotic markers (TGF, CTGF, and collagen-1) and inflammatory cytokines (IL-1 and IL-6) were recorded [15]. Neupogen treatment reinforces innate immunity and enables prevention of inflammation, which is a preliminary step for development of fibrosis [16]. In the current study, there was no significant relation between estradiol administration and IL-1 level, in contrast with that, a down-regulation of the IL-1RtI in uterine epithelial cell line after incubation with estradiol for 72 h was previously reported [17]. Regarding IL-6, the current results revealed that a significant decrease in its expression in MSCs treated groups compared with the fibrosis group and the estrogen treated fibrosis group. Treatment with neupogen alone gives the same results. However, treatment with MSCs + neupogen showed the highest significant decrease in IL- 6 expression levels amongst all the studied groups. Supporting current results, researchers found significant increase in IL-1, IL-6, IL-10, and TNF in a mare model of endometritis with chronic pathological endometrial changes including fibrosis [18]. So, IL-6 causes compromised tissue repair by shifting acute inflammation into a more chronic profibrotic state [19]. Endogenous G-CSF may counter regulate the inflammatory cytokine cascade (IL-1, IL-6, and TNF) and implies a potential indication for filgrastim (recombinant methionyl human G-CSF) in chronic inflammatory conditions [20]. In consistence with the current results, the administration of uMSCs reduced inflammation and inhibited the expression of transforming growth factor-β, and the proinflammatory cytokines (IL- 1 and 6), collagen I, macrophage migratory inhibitory factor, and TNF in bleomycin induced lung fibrosis in mice [21]. Neupogen treatment significantly attenuated hyperoxia-induced lung injury in neonatal rats by down-modulating the gene expression of IL-6, TNF, and TGF [22]. The current results revealed that there was a significant decrease in *TNF* gene levels in the groups treated by MSCs, neupogen, and MSCs + neupogen compared with the untreated fibrosis group. The current results agreed with researchers who found increases in TNF, IL-1β, and IL-6 in bronchoalveolar lavage fluid in pulmonary fibrosis rat model [23]. Estrogen can promote the growth of endometriotic lesions by increasing the TNF levels [24]. BMSCs treatment resulted in the regeneration of the endometrium via modulation of the expression of proinflammatory cytokines TNF and IL-1 in experimental rats [25]. Researchers suggested that neupogen shifts the proinflammatory responses to anti-inflammatory pattern to be used in chronic inflammatory conditions [26].

The current study revealed that there was a significant increase in *VEGF* gene expression in uterine tissues in neupogen, combined MSCs treated groups compared with the fibrosis group.

This work was matched with another work which reported that the immunohistochemical staining of VEGF was increased in endometrial fibrosis rats treated by MSCs alone and MSCs combined with estrogen [8]. Angiogenesis and the hypoxic changes in the endometrial glands and interstitium were also improved in endometrial fibrosis patient treated by hormonal therapy [27]. In consistence with the current results, researchers reported that neupogen increases the serum VEGF by 1.5-fold [28].

The present results revealed that MSCs combined groups showed significant decrease in TGF-β compared with estrogen alone or neupogen alone, which indicates the valuable role of MSCs in limitation of fibrosis though inhibition of TGF-β.

TGF-β cytokines might be involved in formation of IUAs [29]. TGF-β immunoreactivity was high in rats with adenomyosis [30]. A recent study suggested that exosomes derived from human umbilical cord stem cells (hucMSCs) could ameliorate carbon tetrachloride (CCl_4_) induced liver fibrosis through lowering of *TGF-β* gene expression [31]. Hyperstimulation with estrogen increases the expression of TGF-β [32]. Regarding CTGF, there was a statistical significant decrease in neupogen treated group and MSCs treated groups (*P*-value <0.05) with greater significant decrease observed in MSCs and neupogen treated group and decreased fibrosis in histologically examined sections compared with fibrosis group. This agreed with researchers who found that TGF-β and CTGF levels were significantly increased in association with endometrial fibrosis [33]. 17β-estradiol diminishes the development of myocardial fibrosis by decreasing the *TGF-β* and *CTGF* gene expression [34]. *TGF-β* and *CTGF* mRNA were strongly stimulated after estradiol administration in ovarectomized mice [35]. The current results also agreed with researchers who found that MSCs increased VEGF, decreased CTGF, TNF, and IL-6 levels and collagen density in perinatal rats exposed to hyperoxia induced pulmonary dysplasia [36]. Regarding the assessment of collagen I protein by Western blotting, MSCs combined with neupogen significantly decreased the collagen I content compared with all the treated groups. Moreover, neupogen alone significantly decreased the collagen I content compared with the untreated fibrosis group and the treated groups by estrogen alone or MSCs alone. This supported the anti-inflammatory and antifibrotic effect of neupogen. Neupogen is a new therapeutic modality for schistosomiasis through stem cell mobilization, immunomodulation, or fibrosis remodeling [37]. These results support previously reported work which found that the expression levels of fibrotic markers TGF-β, CTGF, collagen I, and collagen III were elevated in IUA rat model [38,39]. In addition, the development of fibrosis in the irradiated lungs was limited after infusion of Ad-MSCs through decreasing the expression of collagen I and collagen III [39]. Estrogen administration alone showed no statistical significant effect to fibrosis group regarding either IL-1, IL-6, TGF-β, CTGF, collagen I levels (inflammatory cytokines or fibrotic factor). Thus, treatment with estrogen alone is not sufficiently an effective therapy. Furthermore, collagen biosynthesis is stimulated by low doses of estradiol in cultured leiomyoma cells [40].

The present results revealed that there was a significant increase in *RUNX* gene expression and antibody in MSCs treated groups either alone or combined with estrogen or neupogen compared with the fibrosis group. RUNX is a novel target for protection against fibrosis-related diseases [41]. RUNX knockout (−/−) mice had loss of leukocytic cell autonomous function and causes inflammatory bowel disease (IBD) [42]. Another study using RUNX knockout mice found that collagen fibers are deposited in a disorganized manner amongst clusters of infiltrating inflammatory cells indicating airway fibrosis [43]. RUNX is required for MSCs cell cycle progression and proliferation and it inhibits their myofibroblastic differentiation [44]. RUNX knockdown models’ study revealed that MSCs exhibited a sharply reduced capacity for proliferation [45].

## Conclusion

From the present study, we can conclude that combined treatment with MSCs and neupogen gives optimum results for reversing the endometrial fibrosis. In addition, estrogen, which is the current treatment of endometrial fibrosis to regenerate the endometrium after surgical removal of IUA, is not a sufficiently effective therapy.

## Abbreviations

CTGF: connective tissue growth factor
G-CSF: granulocyte colony-stimulating factor
hMSC: human mesenchymal stem cell
IL-1: interleukin 1
IL-6: interleukin 6
IUA: intrauterine adhesion
RUNX: runt-related transcription factor
TGF-β: transforming growth factor-β
TNF-α: tumor necrosis factor-α
VEGF: vascular endothelial growth factor
